# Artificial selection on anther exsertion in wild radish*, Raphanus raphanistrum*

**DOI:** 10.1038/sdata.2014.27

**Published:** 2014-09-02

**Authors:** Jeffrey K. Conner, Cynthia J. Mills, Vanessa A. Koelling, Keith Karoly

**Affiliations:** 1 Kellogg Biological Station and Department of Plant Biology, Michigan State University, 3700 East Gull Lake Drive, Hickory Corners, Michigan 49060, USA; 2 National Evolutionary Synthesis Center, 2024 W. Main St, Durham, North Carolina 27705, USA; 3 Biology Department, University of Puget Sound, 1500 N. Warner Street, Tacoma, Washington 98416, USA; 4 Biology Department, Reed College, 3203 SE Woodstock Blvd., Portland, Oregon 97202, USA

## Abstract

To study the genetic architecture of anther exsertion, a trait under stabilizing selection in wild radish, artificial selection on anther exsertion was applied for 11 generations. Two replicate lines each of increased and decreased exsertion plus two randomly-mated controls were included. Full pedigree information is available from generation five. To estimate correlated responses to selection, 571 plants from all lines and matrilines were grown in the greenhouse and a number of floral, growth, and phenology traits were measured. To create an outbred F_2_ mapping population, all possible crosses among the two high and two low exsertion lines were made, using a multiple-family design to capture the genetic variance still present after 11 generations of selection. Six floral traits were measured on 40 parents, 240 F_1_, and 4,868 F_2_ offspring. Opportunities for reuse of these data include traits not previously analyzed, other analyses, especially using the pedigree and fitness data, and seeds from all generations and photos of flowers in the later generations are available.

## Background & Summary

The genetic correlation between filament and corolla tube lengths in wild radish is very high in magnitude (0.85, ref. 1), estimated with precision, and known to be caused by pleiotropy or extremely tight linkage^2^. The relative lengths of these two traits determine the position of the pollen-bearing anthers relative to the opening of the corolla tube; this composite trait is called anther exsertion, which can be defined as ln-long stamen filament length minus ln-corolla tube length. The high filament-corolla tube correlation is likely due to stabilizing selection on anther exsertion by bees in the family Halictidae^3,4^; functionally, intermediate anther exsertion maximizes pollen removal by these bees^5^. Stabilizing selection on the difference between two traits is equivalent to correlational selection to increase the correlation between the traits^6,7^.

**Figure 1:**
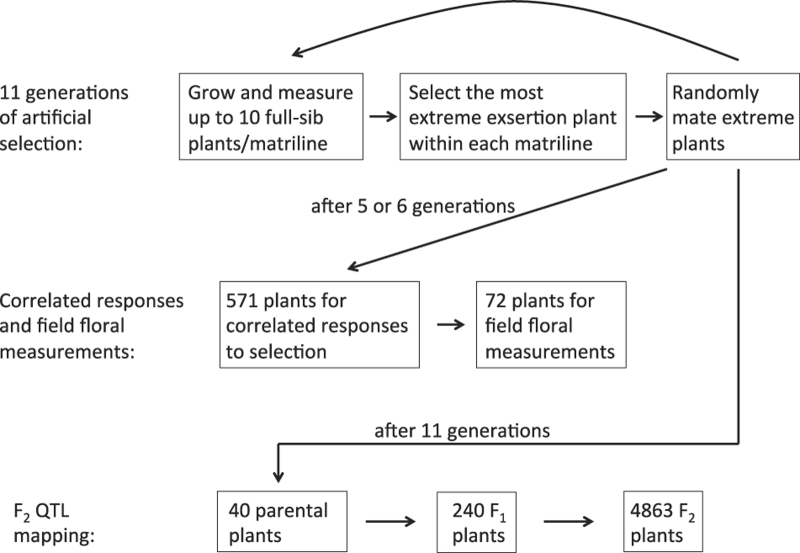
Flowchart of the experiments that produced the datasets. The top three boxes show the procedure used to produce the data in the ‘ArtificialSelectionExsertion.csv’ file, and the other five boxes each correspond to one of the five other datasets.

This paper describes six datasets derived from a series of studies designed to understand selection and genetics of anther exsertion; Figure 1 gives a flowchart of all of the experiments. All plants were derived from a single natural population (see Data Records). To create increased variance in anther exsertion to better test for stabilizing selection, as well as determine the rate of response to selection perpendicular to the major axis of variation, artificial selection for increased and decreased exsertion was performed for a total of 11 generations, with two replicates of each selection treatment^8^. There were also two randomly-mated control lines for a total of six selection lines; each line contained 12 outbred maternal ‘lines’. We refer to these as ‘matrilines’ because they are denoted and followed based on the maternal parent, but each generation these lines were outcrossed using pollen from a unique randomly-chosen plant in a different matriline, so that matrilines are not distinct from each other in the nuclear genome. The floral, fitness, and pedigree data for the four selected lines (not the controls) are contained in the 'ArtificialSelectionExsertion.csv' file.

To test for correlated responses to this selection, after five (replicate 1) or six (replicate 2) generations, 571 plants evenly distributed across the two high, two low, and two control selection lines were grown and 12 floral traits were measured, as well as flowering time and aboveground biomass. These data are in 'CorrelatedResponses.csv'. To quantify floral trait variation over the lifetime of these annual plants, seven floral traits were measured five or six times, and pollen viability was scored twice, over a period of three months on seventy-two of these plants ('2001FieldFlowerMeas.csv').

**Figure 2:**
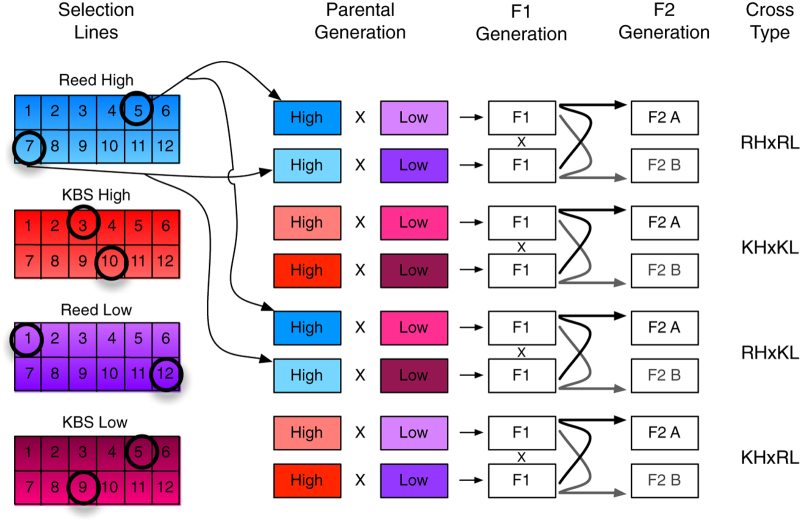
Crossing design for production of 20 outbred F_2_ Families. Shown is the crossing design used for each of the five octets of plants, which produces four full-sib F_2_ families, one for each of the possible high X low exsertion line crosses. Parental plants from selection replicate 1 (Reed) shown in cool colors (blue and purple) and replicate 2 (KBS) in warm (orange and red). The circled individuals depict the plants chosen randomly for this one example octet; arrows to the Parental generation show how the two of these from one line were used in crosses as an example. The crosses in this diagram were repeated five times total in the five octets, using different randomly chosen pairs of parental plants in each line for each; two plants in each line were not used as parents.

An F_2_ mapping population was created by crossing high and low selection lines to determine the genetic basis of the rapid evolution of anther exsertion in the seelction lines. The 11th generation of selection consisted of choosing the extreme anther exsertion plants from 10 matrilines in each of the four selection lines ('QTLParentalMeasurements.csv'). These 40 plants were crossed in all four high by low exsertion line combinations. The resulting F_1_ plants ('QTLF1measurements.csv') were outcrossed to produce 4,863 F_2_ plants distributed among 20 full-sibling families, five per cross type (see Methods; Fig. 2 and Table 1). Six floral traits were measured from floral photographs on each of these F2 plants ('QTL F2 Measurements.csv').

Many analyses of these data are possible in addition to those in the one paper that has been published to date^8^, which used only one of the six datasets included here ('ArtificialSelectionExsertion.csv'). Because anther exsertion and the component traits of filament and corolla tube lengths were measured in 10 generations under selection, multiple times across the lifespan of the same plants, and in a very large outbred F2 composed of full-sibling families from reciprocal crosses means that a variety of questions concerning genetic and microenvironmental causes of trait variation can be addressed. Additional unanalyzed traits are also included in some of the datasets, and photos of the flowers from top and side views are available for additional trait measurements. Novel integrated analyses across these datasets are also a possibility. Stored seeds from virtually all matrilines in virtually all generations are also available for entirely new phenotypic or genomic research.

## Methods

### Artificial selection

We conducted 10 generations (11th in the F2 study below) of selection for increased and decreased long-stamen anther exsertion (ln long filament length—ln corolla tube length), with two replicate lines for each of increased anther exsertion, decreased exsertion, and two randomly-mated controls. Each of the six replicate selection lines consisted of 12 unique matrilines; the most extreme of up to 10 offspring in each matriline was mated in each generation. The matriline of each plant is noted; full pedigree information (paternity) is available starting at generation 5. The first three generations of selection were done at University of Illinois, generations 4 and 5 of replicate 1 of the artificial selection and half of the correlated responses plants were grown at Reed College, and the rest of the greenhouse and lab work at Kellogg Biological Station; thus replicate 1 is also referred to as Reed or R and replicate 2 as KBS or K. For details see ref. 8.

### Outbred F2 QTL design

For future QTL analysis, six plants from each of the 12 matrilines in the two high and two low exsertion selection lines were grown for a total of 288 plants. One flower from each was photographed and the lengths of the corolla tube, short and long filaments, and short and long filament anthers were measured; this was done for all F_1_ and F_2_ plants as well. The plant with the highest or lowest exsertion (matching the selection direction) within each matriline was chosen; this represents the 11th generation of artificial selection on exsertion. This most extreme plant from the 10 most extreme matrilines in each selection line were chosen for the outbred crossing design; the other two matrilines in each selection line were discarded. These 40 parental plants were then randomly paired to make five pairs within each selection line, and then each pair was randomly grouped with a pair from each of the other three lines to form five 'octets' of plants. Each octet was used to produce four outbred full-sibling F_2_ families, one from each of the four cross types; the design for one octet is shown in Figure 2.

To produce the F_1_ generation, each plant was mated to one plant from each of the other lines within the same octet, producing four F_1_ families, one for each of the four possible crosses between high and low exsertion selection lines. Because there were two pairs of parental plants from each selection line, this design produced pairs of unrelated F_1_ plants for each of these four cross types; these pairs were then crossed reciprocally to produce one of the 20 outbred full-sibling F_2_ families (Figure 2). Due to the reciprocal crosses, each of the 20 F_2_ families is subdivided into A and B groups depending on maternal plant.

A total of 4,863 F_2_ plants were grown in 10 blocks of up to 500 plants each, with each full sibling family represented by up to 25 plants per block, and each octet represented by up to 100 plants per block. Blocks alternated between consisting entirely of seeds from the A moms, or entirely of seeds from the reciprocal B moms; thus all the odd number blocks were A seeds, and all the even number blocks B seeds.

## Data Records

The six datasets are stored at Dryad (Data Citation 1). Some contents are common across datasets:

Matriline: All of the plants are descended from the Binghamton NY population (BINY; 42.184089E, 75.835319W) and most have a code with a capital letter A–E and a number up to 475. This refers to the original mothers in the seed collection, where 5 transects (A–E), one meter apart, were run across an alfalfa field and seeds were collected from one maternal plant every meter. The transects varied in length—the last plant collected in each was A368, B385, C355, D475, and E100. The numbers refer to the same grid position in each transect, i.e., B1 is one meter from A1, B2, and C1. Seeds were collected from a total of 1,575 maternal plants, although some have no seeds left. A total of eight matrilines in the high and low replicate 1 populations have different codes without the initial letter; these are descendants from the BINY population but their pedigree cannot be traced back to the original field maternal plant. In a number of cases over the generations a matriline produced no viable seeds, so two families in the next generation came from one matriline; these are denoted with decimals added to the number and/or lowercase letters at the end of the code, but in all these cases the maternal lineage can be traced back to the field maternal plant.

Floral traits: the core set are Petal Length (PetLen), Petal Width (PetWid), Corolla Tube Length (Tube), Short Filament Length (ShrtFil), Long Filament Length (LongFil), and Pistil Length. In the early generations of artificial selection these traits were measured using calipers on dissected flower as described in Conner and Via^1^. In later studies, these are measured from floral photographs, and also include the length of the anther on one short and one long stamen (ShrtAnther and LongAnther). Often the ovules were counted (Ovule#). We often calculated Anther Exsertion as Long Filament minus Corolla Tube. All values are mm.

Treatment: High or H—selection for increased exsertion; Low or L—selection for decreased exsertion; Cntrl—randomly mated controls

Replicate line: 1 (= Reed=R) or 2 (= KBS=K) respectively for the two replicates nested within each Treatment.

Photo: Some files have the code from the camera denoting the image the measurement was made from, available from the first author.

### ArtificialSelectionExsertion.csv

Offspr: the replicate offspring grown from each matriline; in later generations usually 1–10.

ID: a unique integer identifier added in later generations to track the pedigree.

MomID, DadID: the ID of the parents of that plant. In the first generation with IDs, these are lower case letters, because the parents of these individuals were not recorded.

RelFit: Relative fitness=RawFit/Mean Fitness for that line and generation; this is used to estimate selection differentials and gradients.

RawFit: Number of offspring grown and measured in the next generation from that plant. Within each matriline, typically only one will have nonzero fitness, that is, the selected plant, except when different plants within a matriline were used as males versus females due to incompatibility or where a matriline was split due to failure of a different matriline (see above).

Gen: generation of selection.

### CorrelatedResponses.csv:

Matriline, AvPetLen, AvgTube, AvShrtFil, AvgLongFil, Pistil, Ovules: See above, except the four traits with 'Av' were the average of two measurements of different structures within the same flower, i.e., two different petals, filaments, etc. The third flower was measured in most cases, but sometimes a later flower close to the third was used.

Treatment: Direction of artificial selection.

Replicate Line: the two replicates within each treatment.

Block: Plants were grown at KBS or Reed; some traits differed between sites.

CRoffspring#: up to four plants were grown at each location from each matriline

Days to flower: number of days from planting to first open flower.

Nectar vol: volume of nectar in microliters from the 5th and 6th flowers on the central inflorescence.

Nectar conc%: % sugar concentration from refractometry using the same nectar sample.

FlowerNo: The total number of flowers was counted on some plants at Reed at harvest, just over two months after planting.

Biomass: aboveground dry biomass in grams was measured at Reed at harvest.

Total pollen: Number of pollen grains produced were counted using a Coulter Counter on all six anthers from one flower at KBS, and 3 long and 1 short stamen anther at Reed.

LongPollen: the count for the four long stamen anthers at KBS.

ShrtPollen: the count for the short stamen anthers at KBS.

### 2001FieldFlowerMeas.csv

Matriline, AvPetLen, AvgTube, AvShrtFil, AvgLongFil, AvgPistil, AvgOvules: See above, except the traits with 'Av' were the average of the five or six flowers measured over the life of the plant. The individual flower measurements and the date in 2001 that they were taken are in the columns following the averages and denoted by a number 1 through 6 following the variable name.

Treatment: Direction of artificial selection.

Replicate Line: the two replicates within each treatment.

CRoffspring#: up to four plants were grown at each location from each matriline.

DNA ID: The unique code used for the tissue and DNA sample taken from each plant, available from the first author.

Array: These plants were divided into three arrays of 24 plants each; arrays were taken into the field five or six times.

FieldRow and Field Column: The grid positions used for the plants in the field.

AvgFlwr#: The number of flowers open when the plants were taken into the field, averaged over the five or six field days.

### QTL ParentalMeasurements.csv and QTL F1 measurements.csv

All columns as described above except Offspr denotes the six offspring grown from each maternal plant. There are two additional Cross Types in the F_1_ dataset, the High X High and Low X Low; seeds from these are available, but have not been used to make F_2_ plants to date.

### QTL F_2_ measurements.csv

Cross: The four possible crosses between the two replicate high and low exsertion lines—RH=Rep 1 (Reed) High, RL=Rep 1 Low, KH=Rep 2 (KBS) High, KL=Rep 2 Low.

Family: There are five outbred full sib families within each cross; these correspond to the parental 'octets'.

Mom: Crosses of the F_1_ to make each full-sib F_2_ family were done reciprocally, so there is Mom A or B depending on the direction of the cross.

F2: Replicate offspring from each cross. Note that this is redundant with Mom A or B, because all F2s within each family were given a unique number—A is mom for 1–25, 51–75 etc, and B is mom to 26–50, 76–100 etc.

Block: 1–10 for the 10 temporal blocks.

Flwr date: the date that the first flower opened on that plant.

## Technical Validation

**Figure 3:**
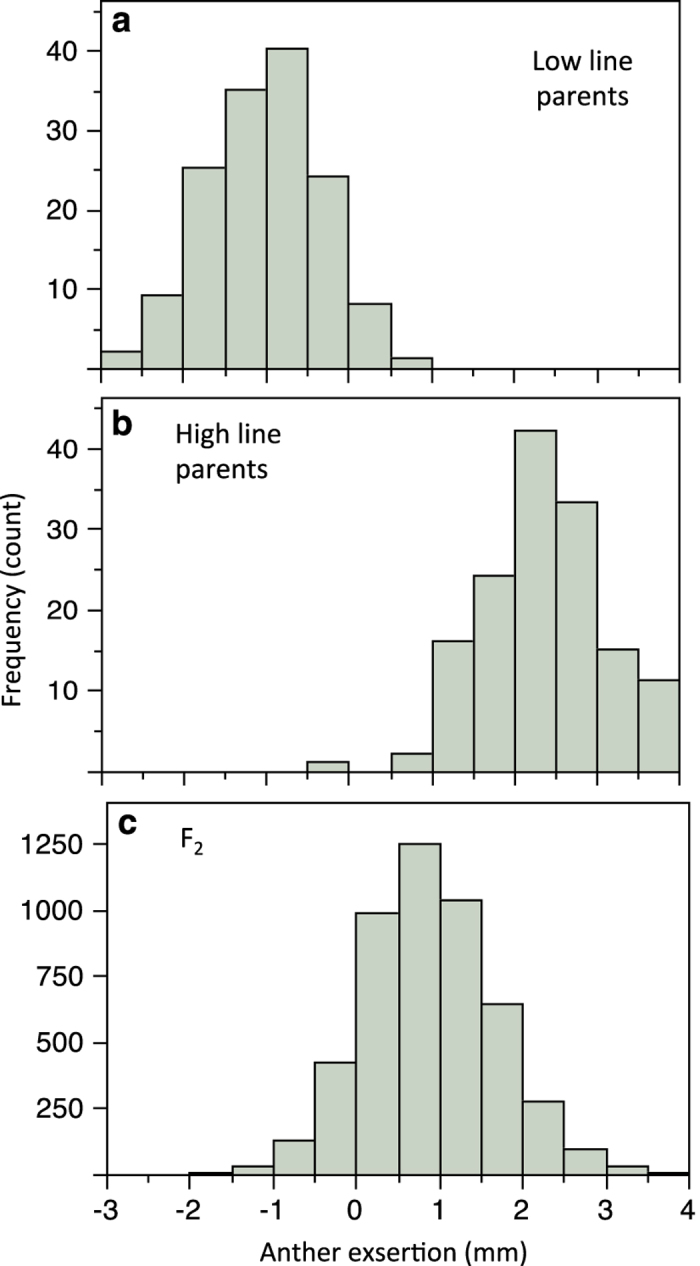
Distributions of anther exsertion from the QTL experiment. Shown are the parents from the low (**a**) and high (**b**) exsertion selection treatments and the F_2_ generation (**c**). In the original data in each case, one plant had values >4 s.d. from the mean; these were found to be simple errors upon remeasuring the original photo and were corrected. Upon remeasurement of the photograph, the one individual with negative exsertion in the high line parents was found to actually have a slightly positive value (0.15).

The distributions of all floral measurements show a good fit to a normal distribution; all outliers (identified graphically as clearly outside the normal distribution) were either validated or corrected using original data or photos (Figure 3). For the artificial selection lines, the very tight fit of the data (R^2^=0.99 for both replicates; Figure 4 in ref. 8) to the fitted regression of response to selection on the selection differential strongly indicates that the data are precise and reliable.

## Additional information

**How to cite this article:** Conner, J. K. *et al.* Artificial selection on anther exsertion in wild radish*, Raphanus raphanistrum*. *Sci. Data* 1:140027 doi: 10.1038/sdata.2014.27 (2014).

## Supplementary Material



## Figures and Tables

**Table 1:** Crosses performed to produce the F_2_ mapping population.

**Female parent**	**Female line**	**Male parent**	**Male line**	**F1 code**	**F2s produced**	**Cross type**
*Octet 1*						
36b/3	RL	A196b/3	KH	KXF1A(1)/2	KXF2(1)	KHxRL
B358a/6	KH	14c/2	RL	KXF1B(1)/2		
A342.2/6	KL	A196b/3	KH	KF1A(1)/3	KF2(1)	KHxKL
B358a/6	KH	D30/3	KL	KF1B(1)/1		
D301/6	RH	A342.2/6	KL	RXF1A(1)/1	RXF2(1)	RHxKL
D30/3	KL	C88.2c/6	RH	RXF1B(1)/4		
36b/3	RL	D301/6	RH	RF1A(1)/2	RF2(1)	RHxRL
C88.2c/6	RH	14c/2	RL	RF1B(1)/3		
*Octet 2*						
C135/1	KH	D367a/6	RL	KXF1A(2)/1	KXF2(2)	KHxRL
E74.1a/1	RL	B358b/3	KH	KXF1B(2)/4		
A327b/6	KL	C135/1	KH	KF1A(2)/4	KF2(2)	KHxKL
B358b/3	KH	C47.1d/4	KL	KF1B(2)/1		
C88.2b/4	RH	A327b/6	KL	RXF1A(2)/2	RXF2(2)	RHxKL
C47.1d/4	KL	A209d/3	RH	RXF1B(2)/1		
D367a/6	RL	C88.2b/4	RH	RF1A(2)/3	RF2(2)	RHxRL
A209d/3	RH	E74.1a/1	RL	RF1B(2)/3		
*Octet 3*						
B66.1d/6	RL	A343/5	KH	KXF1A(3)/4	KXF2(3)	KHxRL
C90/2	KH	E72/1	RL	KXF1B(3)/2		
B189.1d/4	KL	A343/5	KH	KF1A(3)/1	KF2(3)	KHxKL
C90/2	KH	C46.1/1	KL	KF1B(3)/1		
C88.2a/1	RH	B189.1d/4	KL	RXF1A(3)/1	RXF2(3)	RHxKL
C46.1/1	KL	C180c/6	RH	RXF1B(3)/2		
B66.1d/6	RL	C88.2a/1	RH	RF1A(3)/3	RF2(3)	RHxRL
C180c/6	RH	E72/1	RL	RF1B(3)/3		
*Octet 4*						
D466/5	KH	C127.1d/6	RL	KXF1A(4)/1	KXF2(4)	KHxRL
1d/3	RL	C214b/2	KH	KXF1B(4)/1		
E62.1b/1	KL	D466/5	KH	KF1A(4)/1	KF2(4)	KHxKL
C214b/2	KH	B189.1a/4	KL	KF1B(4)/1		
C180b/2	RH	E62.1b/1	KL	RXF1A(4)/1	RXF2(4)	RHxKL
B189.1a/4	KL	A209a/3	RH	RXF1B(4)/1		
C127.1d/6	RL	C180b/2	RH	RF1A(4)/3	RF2(4)	RHxRL
A209a/3	RH	1d/3	RL	RF1B(4)/1		
*Octet 5*						
E93d/3	RL	D171/2	KH	KXF1A(5)/1	KXF2(5)	KHxRL
C214a/1	KH	36d/6	RL	KXF1B(5)/1		
C47.1e/2	KL	D171/2	KH	KF1A(5)/1	KF2(5)	KHxKL
C214a/1	KH	E62.1a/5	KL	KF1B(5)/2		
C97/6	RH	C47.1e/2	KL	RXF1A(5)/2	RXF2(5)	RHxKL
E62.1a/5	KL	50c/3	RH	RXF1B(5)/2		
E93d/3	RL	C97/6	RH	RF1A(5)/1	RF2(5)	RHxRL
50c/3	RH	36d/6	RL	RF1B(5)/3		
The unique codes for all plants used in parental and F_1_ crosses are given, plus the selection line the parents came from, the 'octets' the parents were assigned to, and the Cross Type that appears in the F2 measurements file. The offspring number (given in the Parental and F_1_ measurements files) from each parental and F_1_ maternal plant that was used in the cross is given after the forward slash (/).						
